# Soil Metagenomes from Different Pristine Environments of Northwest Argentina

**DOI:** 10.1128/genomeA.00926-15

**Published:** 2015-08-13

**Authors:** Christina B. McCarthy, Déborah I. Colman

**Affiliations:** a Centro Regional de Estudios Genómicos, Facultad de Ciencias Exactas, Universidad Nacional de la Plata, La Plata, Buenos Aires, Argentina; b Departamento de Informática y Tecnología, Escuela de Ciencias Agrarias, Naturales y Ambientales, Universidad Nacional del Noroeste de la Provincia de Buenos Aires, Pergamino, Buenos Aires, Argentina

## Abstract

This is the first study to use a high-throughput metagenomic shotgun approach to explore the biosynthetic potential of soil metagenomes from different pristine environments of northwest Argentina. Our data sets characterize these metagenomes and provide information on the possible effect these ecosystems have on their diversity and biosynthetic potential.

## GENOME ANNOUNCEMENT

Soil microbiota produce many of the most important pharmaceutical drugs, including antibiotics and cancer drugs (1). Nevertheless, the traditional approach for characterizing the biosynthetic capacity of environmental bacteria, i.e., culturing them in the laboratory, has provided access to only a small fraction of this potential (2, 3). Recent analyses of soil microbiomes from around the world revealed a vastly unexplored biosynthetic diversity which was associated with soil types (4–7). In general, arid soils showed the richest biosynthetic diversity (5) and, similarly, bacterial diversity was highest in neutral soils (generally arid and semiarid ecosystems) and lower in acidic soils (generally tropical forest ecosystems) (7).The purpose of this study was to characterize soil metagenomes from different pristine environments using a metagenomic shotgun approach, giving special emphasis to the biosynthetic potential of each soil type. For this, four soil samples collected in northwest (NW) Argentina were analyzed. Sampling sites were chosen at different altitudes from the Yungas (YU) and Argentine Northwest Monte and Thistle of the Prepuna (NWMT) regions, with soils of varying pHs, namely: 1) YU (Montane Forest District) at 1,500 m above sea level (MASL) in Tafí del Valle (Tucumán, Argentina) (named Soil_TV; S27°01.123′; W65°39.807′; pH 5.35); 2) YU (Montane Cloud-forest District) at 850 MASL in Rosario de la Frontera (Salta, Argentina) (Soil_RF; S25°50.143′ W64°55.524′; pH 8.01); 3) NWMT at 1,600 MASL in Cafayate (Salta) (Soil_CA; S26°03.885′ W65°56.506′; pH 7.05); and 4) NWMT at 1,600 MASL in Quebrada de las Conchas (Cafayate Department, Salta) (Soil_QC; S26°01.123′ W65°49.429′; pH 8.92). For the extraction of DNA, the three samples that contained more organic material (Soil_TV, Soil_RF, and Soil_CA) were processed with the QIAamp stool minikit (Qiagen), whereas Soil_QC was processed according to reference 8, treated with RNase (Invitrogen), and precipitated with LiCl and ethanol. High-throughput pyrosequencing of the samples was performed using a Roche GS FLX (Macrogen, Inc., South Korea), yielding ~1.15 Gb of metagenomic reads with lengths of 40 to 1,074 bases (nt) (520 nt average).

Raw sequence reads were trimmed using a custom application for removing nucleotides derived from the amplification primers (9, 10), and then processed with CD-HIT-454 (11). The nonredundant protein sequence NCBI database (DB:nr) was downloaded locally, and RAPSearch2 (12) was used to perform the protein homology search of the trimmed clustered reads against DB:nr. The taxonomic and functional content of the data sets was then analyzed with MEGAN (13, 14). Metagenomes consisted of 65.6% to 61.5% bacteria, 1.9% to 0.36% archaea, 1.6% to 0.17% eukaryota, and 0.1% to 0.01% viruses. Statistical analysis (*P* < 0.05, Fisher’s exact test [15]) indicated significant differences between all samples. Diversity (Shannon-Weaver index) was highest in Soil_CA, followed by Soil_RF and Soil_TV, whereas Soil_QC showed the lowest diversity.

This is the first study to use a metagenomic shotgun approach to generate soil metagenome data sets from different pristine environments of NW Argentina. These data sets indicate the presence of bacteria, archaea, eukaryota, and viruses in all the samples and provide information on the potential effects of ecosystem types (including pH and altitude) on the composition, diversity, and biosynthetic potential of these soil metagenomes.

### Nucleotide sequence accession numbers.

Nucleotide sequences were submitted to the NCBI Sequence Read Archive (SRA) under the accession numbers SRX1058163, SRX1058164, SRX1058165 and SRX1058166.
